# Massage therapy in infants and children under 5 years of age: protocol for an overview of systematic reviews

**DOI:** 10.1186/s13643-021-01681-x

**Published:** 2021-04-28

**Authors:** Shu-Cheng Chen, Juan Yu, Sam Chun-Sum Yuen, Jason Chun-Sing Lam, Lorna Kwai-Ping Suen, Wing-Fai Yeung

**Affiliations:** 1 School of Nursing, Hong Kong Polytechnic University, 11 Yuk Choi Road, Hung Hom, Kowloon, Hong Kong SAR, China; 2 Pediatric Tuina Health Care Clinic, Shandong University of Traditional Chinese Medicine Affiliated Hospital, Jinan, China; 3 School of Chinese Medicine, Hong Kong Baptist University, Hong Kong SAR, China; 4 School of Pharmacy, the Chinese University of Hong Kong, Hong Kong SAR, China; 5 School of Nursing, Tung Wah College, Hong Kong SAR, China

**Keywords:** Massage, External therapy, Infants and children under 5 years, Paediatric condition, Overview, Systematic review

## Abstract

**Introduction:**

Massage is a popularly used complementary and alternative therapy. Previous randomised controlled trials have examined the effects of massage on children, and several systematic reviews have been conducted to synthesise these data. This study aims to assess and summarise the current evidence from published systematic reviews of controlled clinical trials on the practice of paediatric massage, specifically in infants and children aged < 5 years.

**Methods:**

The online databases MEDLINE, Embase, Health Technology Assessment Database, Cochrane Database of Systematic Reviews, Database of Abstracts of Reviews of Effects, Allied and Complementary Medicine, China National Knowledge Infrastructure and Wanfang Data will be searched from the inception onwards for evidence of the treatment effects. We will include systematic reviews of randomised control trials evaluating the effects and safety of massage therapy in infants and children aged < 5 years. The primary outcomes will be any physical or psychological outcome, and adverse effects on children. Secondary outcomes will include any physical or psychological outcome on caregivers. Two reviewers will independently screen the articles for inclusion as per the eligibility criteria. They will extract information from the included studies and assess the methodological quality of the included studies. A table will be used to summarise of information of the included studies, which includes the basic information, method and findings. The methodological quality of the included systematic reviews will be assessed by A Measurement Tool to Assess Systematic Reviews version 2 (AMSTAR 2). Extracted data from the included studies will be collected and presented using narrative approach. The pooled effect estimates for meta-analysed outcomes will be extracted when possible. If there is a discrepancy in results of two or more reviews on the same topic, then the causes of such discrepancy will be further explored.

**Discussion:**

This overview of systematic reviews will summarise the current evidence on massage, specifically for infants and children aged < 5 years. We will comprehensively present the positive effects and adverse effects of this intervention. Findings from this overview will be published in a peer-reviewed journal.

**Systematic review registration:**

CRD42020186003.

**Supplementary Information:**

The online version contains supplementary material available at 10.1186/s13643-021-01681-x.

## Background

Child (aged < 5 years) and infant health is a major public health concern worldwide [1]. Certain medical conditions, such as preterm complications, diarrhoeal diseases and congenital muscular torticollis, will greatly impair infant and child development if not properly treated. For example, the complications of premature birth may lead to cerebral palsy, impaired learning, vision and hearing problems, behaviour and psychological problems and chronic health issues [2]. Patients with congenital muscular torticollis symptoms or comorbidities usually suffer from cosmetic problems and other associated physical dysfunctions [3, 4]. Diarrhoeal disease is the second leading cause of death and malnutrition in children aged < 5 years [5]. Therefore, timely diagnosis and interventions are crucial in treating and improving these paediatrics conditions [6].

Although evidence-based treatments are available, the use of complementary and alternative medicine (CAM) has been increasingly popular among paediatric patients worldwide. In the USA, 11.8% of children have used some form of CAM in 2007 [7], whereas a survey in 2016 showed that 20% of households with children in Canada used CAM therapies [8]. It has been reported that the 12-month prevalence of CAM usage in children was 18.4% in Australia [9]. The prevalence of CAM therapies was higher in Asia and Europe, where the mean prevalence was 45% across 20 European countries [10] and approximately 65.3% in Korea [11]. In general, children are more likely to receive CAM treatment due to parents’ prior experience and positive perception of CAM [12]. Parents choose CAM therapies over conventional medicine for their children because a particular CAM treatment was considered effective and due to fear of side effects of medications, dissatisfaction with conventional medicine and the need for more personal attention [13]. Medications such as antibiotics and chemotherapeutics can induce toxicities and have negative influences on the enteric immune system in children and adults [14]. CAM modalities, such as skin-to-skin contact and mind-body therapies, are often necessary in paediatrics, due to their relative safety, affordability and ease of implementation. Parents are more likely to use CAM for strengthening the immune system, physical stabilisation and maintaining the health of their children [7, 15, 16].

### Massage therapy

Among the different CAM modalities, massage therapy is one of the most commonly used in the paediatric population. Massage is a complementary and alternative therapy that involves manipulating the soft tissues of the body for improving health conditions [17]. Massage for infants and children requires unique approaches (e.g. manipulations, frequency, locations, strength, permission of children), which determine the effects of this intervention [6, 18]. The prevalence of use of massage in children among all types of CAM ranged from 8 to 25% in UK, USA, Australia and Canada [8, 9, 19, 20]. Massage therapy is popular in the treatment of particular health conditions in children, such as cancers, pulmonary disease and sickle cell disease [21]. Massage therapy was ranked the second most common CAM therapy in two studies [21, 22]. In a survey on the prevalence of massage for cerebral palsy, 80% of the children were found to have received this therapy [23]. In general, massage therapies applied to children are collectively known as paediatric massage, and the application of pediatric massage is usually based on life experience or clinical cases. Some paediatric massage types have their specific theoretical system, such as paediatric *tuina*, which is an independent modality of traditional Chinese medicine (TCM) therapies and is based on TCM meridian theories. This paediatric massage type works well in infants and children aged < 6 years, especially those aged < 3 years [6]. Massage has been used in many paediatric conditions, such as abdominal pain [24], diarrhoea [25], constipation [26], anorexia [27], autism spectrum disorder [28], attention deficit hyperactivity disorder [29], eczema [30], preterm complications [31], asthma [32] and congenital muscular torticollis (CMT) [33]. Until now, there has been no established international treatment guideline on the usage of massage therapy in children.

The mechanisms of massage are still not fully understood. Some researchers believe that massage might help promote the body to heal itself and return to homoeostasis. The receptors in the skin detect a range of stimuli, such as light touch or pressure, and transmit the signals from the periphery to synapses in the central nervous system. Subsequently, the brain integrates these signals into effective actions via the regulation of the neuroendocrine-immune network [34]. Some researchers put forth that the gate control theory may explain the mechanism of massage for pain management. According to this theory, massage increases large nerve fibre (fibres that carry sensations of pressure and touch to the spinal cord) activity, which inhibits the effects of small nerve fibres (fibres that carry pain signals to the spinal cord) [35]. Massage could correct deformity in conditions of the musculoskeletal system through manipulations in specific directions. For example, infants with CMT are strongly recommended to receive stretching manipulation in the reverse direction to the atypical posture to elongate shortened muscles as long as they are identified [36]. Previous clinical trials suggested that massage has potential effects on many paediatric conditions, such as anxiety [37], pain [38], sleep disturbance [39], gastrointestinal functioning [40], immune functioning [41], cognitive problems [42] and emotional disorders [43]. A number of systematic reviews have also been conducted to gather evidence of massage therapy in paediatrics [27, 44–46]. Field conducted a narrative review on summarising the literature on massage therapy in the past decade and showed that this intervention may have beneficial effects on many paediatric conditions [47]. Although massage is commonly used in infants and children with various conditions, the evidence of the therapeutic effects is not strong enough to support its usual use due to the biases of many previous studies. Furthermore, there has been no systematic overview, specifically on infants and children aged < 5 years. Therefore, we designed this overview of systematic reviews to further summarise the existing evidence of massage for the conditions of infants and children aged < 5 years.

### Aims

This overview of systematic reviews aims to summarise the existing evidence on the treatment effects and safety of massage therapy in infants and children aged < 5 years.

## Methods

This protocol of overview of systematic reviews is being reported in accordance with the Preferred Reporting Items for Systematic Reviews and Meta-Analysis Protocols (PRISMA-P) [48] (see checklist in Additional file 1). This protocol has been registered within the International Prospective Register of Systematic Reviews (PROSPERO) database (registration ID: CRD42020186003).

Overviews refer to systematic reviews of systematic reviews in this research [49]. We will search for and identify multiple systematic reviews on paediatric massage-related study questions explicitly and systematically to extract and analyse the results across significant outcomes. The methods have been chosen in consultation with the methodological work of Cochrane Handbook (Cochrane Handbook, Chapter V.4). We will consider published articles of completed systematic reviews of randomised controlled trials. We will include reviews that explicitly stated methods to identify studies (e.g. a search strategy), explicitly stated methods of study selection (e.g. criteria for inclusion) and explicitly described methods of qualitative and/or quantitative data synthesis. We will also perform a supplementary systematic review to summarise the evidence of the adverse effects of massage on infants and children aged < 5 years. The methods of this review are based on the criteria of conducting systematic reviews of adverse events in the *Cochrane Handbook of Systematic Reviews of Interventions* [50], and the *CRD*’*s guidance for undertaking reviews in health care* [51]. 

### Criteria for considering reviews for inclusion

The eligibility criteria for the clinical question guiding this overview are presented in the PICOTSS (patient, intervention, comparators, outcomes, timing, setting and study design) format (Table 1).

**Table 1:** Eligibility criteria using the PICOTSS (Patients, Interventions, Comparators, Outcomes, Timing, Setting, and Study Design) format

**Patients** • Infants and children aged < 5 years	
**Intervention** • Massage therapy	
**Comparators** • Waitlist control • Placebo • Positive controls • Pharmacological treatments • Combinations of treatments • Usual care • Standard care	
**Outcomes** • *Children* ▪ Physical outcomes (e.g. pain assessment, pulse rate, length of hospital admission, readmission) ▪ Psychosocial outcomes (e.g. anxiety, depression, insomnia, fears, behavioural regression) ▪ Developmental outcomes (e.g. weight gain, intelligence, ability of learning knowledge) • *Caregivers* ▪ Physical outcomes (e.g. blood pressure) ▪ Psychological outcomes (e.g. economic pressure, anxiety, depression) • *Safety of interventions* ▪ All adverse events (e.g. an increase in anxiety after receiving an intervention, high drop-out rate)	
**Timing** • Studies of any duration	
**Setting** • Studies of any setting	
**Study design** • Systematic reviews with randomised controlled trials	

### Types of studies

We will include published systematic reviews and randomised controlled trials (RCTs).

### Types of participants

We will include systematic reviews in which participants are infants and children aged < 5 years.

### Types of interventions

We will include systematic reviews that examine the effects and/or safety of massage therapy for infants and children aged < 5 years. Massage is defined as manipulations conducted on the soft tissue of a child’s body, which might include kneading, grasping, pressing, pushing, nipping, rotating, stretching, rubbing and so forth [52]. This intervention can be used for treating or preventing diseases, improving the situation of medical conditions, promoting growth, health preservation, improving immunity, among others. We will include all types of massage therapies.

### Types of comparisons

We will include systematic reviews of RCTs that compare the effects between massage therapy and other interventions, control interventions, or no intervention. The comparisons include waitlist control, placebo or sham controls, positive controls, pharmacological treatments, combinations of treatments and usual or standard care. Studies comparing the effects of different kinds of massage therapies, or between massage and other complementary and alternative therapies, will be excluded.

### Types of outcomes

The primary outcomes will be any physical and psychological outcome reported by children and/or parents, and adverse effects on children.

- Physical outcomes (e.g. blood pressure, pain assessment, pulse rate, weight change, length of hospital admission, readmission)
- Psychological outcomes (e.g. anxiety, depression, insomnia, fears, behavioural regression, self-esteem, mood, fears and month-child attachment)
- Adverse events (e.g. an increase in anxiety after receiving an intervention, high dropout rate) [50]. Other secondary outcomes are parents’ or caregivers’ physical (e.g. blood pressure, pulse rate, weight change) or psychological outcomes (e.g. stress, anxiety, depression, self-esteem, mood, fears) 

### Timing

We will include systematic reviews of RCTs of any duration, treatment period and treatment frequency.

### Setting

We will include systematic reviews of RCTs that have been conducted in any setting. The intervention could be implemented anywhere, such as hospitals and at home. The therapists could be parents, caregivers, paediatricians, experts, practitioners or other qualified personnel. The clinical trials could be pilot studies, feasibility studies, fully powered studies or other phases. There are no limitations in other aspects of the setting.

### Information sources and search strategy

We will search the following databases, from inception onwards: Ovid MEDLINE, Embase, Health Technology Assessment Database (HTA), the Cochrane Database of Systematic Reviews, Database of Abstracts of Reviews of Effects (DARE), Allied and Complementary Medicine (AMED), China National Knowledge Infrastructure (CNKI) and Wanfang Data. The following keywords will be used: (massag* OR touch OR tactile stimulation OR anmo OR acupressure OR *tuina* OR manipulat*) AND (newborn* OR child* OR baby OR babies OR infant* OR youth OR paediatric* OR paediatric* OR toddler* OR pre-school* OR pre-school*). The literature searches will be designed and conducted by the review team in consultation with a health information specialist. The search will include a broad range of terms and keywords related to massage, children and systematic reviews. A draft search strategy for EMBASE is provided in Additional file 2. To search the Chinese databases, the corresponding Chinese keywords will be used. We will contact the authors of the studies if necessary information is missing from the publications. References for the included studies will be searched for useful information. To address the on-going systematic reviews, we will also search the PROSPERO database to identify the registered relevant systematic reviews. We will not impose any time or language restrictions. Information on adverse effects from the included systematic reviews may not be comprehensive. To supplement this, the index term, free-text searching approach and ‘Grey’ literature hand-searching will be used for identifying articles with information on adverse effects.

### Data collection and analysis

#### Selection of reviews

Two authors (SCC and CSY) will independently screen the results of the electronic search by title and abstract. For both objectives, we will obtain the full-text report of the systematic reviews and studies deemed appropriate and uncertain, and subsequently apply the PICOTSS eligibility criteria to determine the final inclusion list. Studies that do not meet the inclusion criteria will be excluded. We will resolve disagreements between review authors through discussion or consultation with an additional reviewer (WFY) when necessary. We will provide a PRISMA flow diagram documenting the screening and review the selection process.

#### Data extraction and management

Two authors (SCC and CSY) will independently extract data in duplicate using a standard data extraction form. Discrepancies will be resolved by discussion or consultation with an additional reviewer (WFY) if necessary. If specific data are missing or inadequate, we will contact the original systematic review authors to obtain or confirm the information. Data extraction form will include the following details:

1. Systematic review basic information (first author, year of publication, number of studies and participants included in the systematic review)
2. Systematic review characteristics (reported strategies to search literature, number of databases searched and date of last search, any restrictions (e.g. language, geographic or date), objective(s), inclusion/exclusion criteria, intervention(s) of interest and comparators, target condition, patient population, main outcomes of interest, type of study designs included, number of studies reporting data for meta-analyses (when appropriate), effect metric(s) reported (e.g. risk ratio), methods to assess study risk of bias, statistical methods to combine studies
3. Finding summary: meta-analytic estimates including heterogeneity measures, additional analyses (e.g. subgroup analysis, sensitivity analysis, or meta-regression), assessment of methodological and risk of bias (e.g. publication bias across studies), GRADE judgements regarding the quality of evidence where present, funding source and conflicts of interest.

### Assessment of the methodological quality of the included reviews

Two authors (SCC and CSY) will be responsible for the methodological assessment. Discrepancies will be resolved by consensus between the two authors or by consulting a third reviewer (WFY) if necessary.

According to the requirement of the Cochrane handbook, both the methodological quality of the reviews included and the evidence quality of the individual studies included in the reviews must be assessed.

#### Quality of the included reviews

The methodological quality of the included systematic reviews will be assessed using the AMSTAR 2 (A Measurement Tool to Assess systematic Reviews 2) instrument [53, 54]. AMSTAR 2 assists in evaluating the quality of conducting systematic reviews using 16 distinct items, 7 of which are critical domains that can have an impact on the validity of a systematic review. The seven critical domains include protocol registration before the commencement of the review (item 2), comprehensiveness of the literature search strategy (item 4), justification of exclusion (item 7), risk of bias of the studies included in the reviews (item 9), appropriateness of meta-analytical methods (item 11), consideration of risk of bias when interpreting the results of the review (item 13), and assessment of the presence and likely impact of publication bias (item 15). Each AMSTAR item will be rated as ‘Yes’ (clearly done) or ‘No’ (clearly not done or without information) in light of the published systematic review. Some items provide a ‘partial Yes’ for responding to the situations that we think are worthy of partial adherence to the criteria. AMSTAR 2 assists users in identifying the potential influence of flaws or weaknesses in each domain. Quality of systematic reviews will be rated as ‘High’ (no or one non-critical weakness), ‘Moderate’ (more than one non-critical weakness), ‘Low’ (one critical flaw with or without non-critical weaknesses) or ‘Critical low’ (more than one critical flaw with or without non-critical weaknesses) [54].

#### Quality of evidence in the included reviews

We will assess the risk of bias of the individual trials included in the systematic reviews using the Cochrane collaboration’s tool (RoB 2) [55], which assesses the risk of bias of randomised trials in six domains as ‘Low risk’, ‘Unclear risk’ and ‘High risk’. These domains are selection bias, performance bias, detection bias, attrition bias, reporting bias and other bias. The overall quality of evidence across studies for each significant outcome will be assessed using the Grading of Recommendations Assessment, Development and Evaluation (GRADE) approach [56]. The level of evidence will be determined by assessing the following aspects of the studies: number of studies, study design, risk of bias, inconsistency of the findings, imprecision, indirectness of the estimate and other considerations, such as publication bias. A risk of bias (RoB) table and a summary of findings (SoF) table based on the methods described in the Cochrane Handbook for Systematic Reviews of Interventions will be built up to convey key information on the effects of massage intervention for each condition and the overall credibility of the information [49].

### Data synthesis

We will report the results according to the Cochrane Handbook of Systematic Reviews of Interventions. A PRISMA flowchart will be used to present the process of study selection for both the overview and the systematic review. We will summarise the evidence for each universe of diseases, disorders, or other conditions. The universes will be classified according to the International Classification of Diseases, 11th version (ICD-11), which is the international standard for reporting diseases and health conditions [57].

Characteristics of the included systematic reviews such as key features, effect estimates, 95% confidence intervals and measures of heterogeneity (if studies are pooled), as well as findings and variations of the studies will be extracted, transformed and presented in tables, and graphics (e.g. funnel plots) will be used as appropriate.

If multiple reviews/meta-analyses include information from overlapping individual studies, a meta-analysis will not be performed. Instead, a qualitative synthesis or summary for each outcome and intervention reporting the pooled treatment effects from the most comprehensive and highest-quality meta-analyses (as assessed by the AMSTAR-2 approach). We will provide a narrative summary of the quality assessment of the included meta-analyses, which will be supported by a table showing the results of the critical appraisal.

If there is discrepancy in the results of these reviews, then we will further explore the factors that lead to the variations or discrepancies, such as eligibility criteria settings, literature search details, outcome definitions used and statistical analysis approaches.

The problem of double-counting data will be considered prudently. To minimise the reduplicative information extracted from overlapping trials, the following strategies will be applied:

- If the qualities of these reviews are similar, we will select the one that contributes the most outcome data.
- If the outcomes of these reviews are completely overlapping, we will retain the one with the highest quality.
- If the outcomes of these reviews are partly overlapping, we will completely retain the highest-quality review and partly retain those with lower-quality.
- If the outcomes of these reviews do not overlap, we will retain all.
- If the outcomes of these reviews are completely overlapping and their qualities are similar, we will select the most recent.

We will present the effects of massage therapy based on the most comprehensive reviews with the highest quality. For the overlapping reviews that are not included in the intervention effects analysis, we will report their general information in a table to enable readers to obtain useful data. The AMSTAR 2 instrument, Cochrane RoB tool and GRADE approach will be used to assess the quality of the reviews.

When possible, we will extract and report pooled effect estimates for meta-analysed outcomes for each review that meets the inclusion criteria. For dichotomous outcomes, relative risks (RRs) with 95% confidence intervals (CIs) will be pooled, while for continuous outcomes, mean differences (MDs) with 95% CIs for the same outcome measure or standardised mean differences (SMDs) with 95% CIs for different outcome measures will be expressed. For time-to-event data, hazard ratios (HRs) with 95% CIs will be expressed [49]. However, we will not compute an overview meta-estimate due to the heterogeneity in ages and outcomes between trials, the absence of essential data and the lack of well-established quantification methods.

Adverse effects of massage therapy reported in the systematic reviews will be listed and summarised narratively. The mechanism of adverse effects of therapies for several conditions might be similar in different populations and settings [50]. Therefore, we will collect adverse effects regardless of the condition or how massage therapy was conducted. We will also collect information on adverse effects from the overlapping included systematic reviews. We will consider a high drop-out rate (≥ 20%) as an outcome measure in study reports for adverse effects, since withdrawal might be related to upsetting side effects, stress on subjects, or others [49, 58].

According to the results synthesised from the data on effects and safety issues, we will generate a figure to present the recommendation level of massage therapy for each included condition, considering the gender, age, and other factors. Since the mechanism of treatment effects might be similar on the same outcomes across different conditions, we will also generate a figure to summarise evidence for each outcome, if possible. Sufficient systematic review evidence will be the most important criterion for the generation of the final recommendation level figure, and all adverse effects for each condition will be emphasised and marked.

## Ethics and dissemination

Results of this overview will be published in a peer-reviewed academic journal and presented at relevant national or international conferences. The study does not need ethical approval since it will not collect individual information.

## Discussion

This overview will provide comprehensive evidence of massage therapy for infants and children aged < 5 years. We will present the treatment effects and adverse effects of massage therapy both quantitatively and qualitatively, if possible. The high mortality rate of infants and children aged < 5 years is a global public health concern. Our results may provide useful information for patients, care givers, healthcare workers, paediatricians and policy makers.

There will be a specific summary of adverse effects in the overview of systematic reviews to achieve balanced perspectives in accessing massage therapy. This is based on the requirements of the Cochrane handbook, indicating that all reviews ought to consider the adverse effects of an intervention. A summary of the adverse effects of massage therapy is significant since, compared to patients of other age groups, infants and children aged < 5 years are more sensitive to all interventions, and they have difficulties in expressing their feelings. Therefore, the highest priority ought to be given to safety when this age group receive interventions.

Some practical issues may occur during this study process. First, there might be some outcomes which are not validated in the reviews. For example, ‘effective rate’, which is usually defined as the proportion of patients with at least some improvement, is usually used for assessing the improvements of an intervention in the clinical trials in China [59]. However, the effective rate is not standardised, making comparison with other validated outcome measures not possible. We will consider the practicability of the emerging additional outcomes for the clinical professionals and policy makers, and then decide whether the outcomes could be included by discussion. Second, some eligible systematic reviews might contain data not only about infants or children aged < 5 years but also for older children and adolescents. This may affect the pooled effects. We will further explore the impact of these studies using sensitivity analyses.

There are some limitations to this overview of systematic reviews. First, the depth of this review depends on the availability of the current literature. We have included comprehensive search terms and hand-search of relevant reviews to exhaust the current evidence. Secondly, we will only include articles in English and Chinese, which might lead to missing relevant data in other languages. Thirdly, the quality of the original studies might vary in several aspects, such as random sequence generation, allocation concealment, selective data reporting, blinding and adverse event monitoring, which might potentially undermine the reliability of the systematic reviews, and have chances to affect the findings of the future umbrella review.

If the protocol is significantly revised in the future, we would document the amendments.

Dissemination of the findings of the overview will be carried out via submitting to the peer-reviewed journals, presenting on academic conferences, providing online or face-to-face courses for clinical therapists or parents and being demonstrated the results on social media sites.

## Supplementary Information

**Additional file 1.** PRISMA-P (Preferred Reporting Items for Systematic review and Meta-Analysis Protocols) 2015 checklist: recommended items to address in a systematic review protocol.

**Additional file 2.** Embase Search strategy.

## Data Availability

Not applicable.

## Abbreviations

CAM: Complementary and alternative medicine
TCM: Traditional Chinese medicine
CMT: Congenital muscular torticollis
RCT: Randomised controlled trial
HTA: Health Technology Assessment Database
DARE: Database of Abstracts of Reviews of Effects
AMED: Allied and complementary medicine
CNKI: China National Knowledge Infrastructure
GRADE: Grading of Recommendations Assessment, Development and Evaluation
ICD: International Classification of Diseases
RR: Relative risk
CI: Confidence interval
MD: Mean difference
SMD: Standardised mean differences
HR: Hazard ratio
