# Supplementary material for: Massage therapy in infants and children under 5 years of age: protocol for an overview of systematic reviews
Source: Syst Rev. 2021 Apr 28;10:127. doi: 10.1186/s13643-021-01681-x (PMC8082656; doi:10.1186/s13643-021-01681-x)
Supplement: Supplementary file 2 — Additional file 2. Embase Search strategy. [file 13643_2021_1681_MOESM2_ESM.docx]

| Additional file 2. Embase Search strategy | |
| --- | --- |
| Code | **Keywords** |
| 1 | (massag* OR touch OR tactile stimulation OR anmo OR acupressure OR tuina OR manipulat*).mp |
| 2 | exp massage/ |
| 3 | exp tactile stimulation/ |
| 4 | exp acupressure/ |
| 5 | exp manipulative medicine/ |
| 6 | 1 or 2 or 3 or 4 or 5 |
| 7 | (newborn* OR child* OR baby OR babies OR infant* OR youth OR pediatric* OR paediatric* OR toddler* OR preschool* OR pre-school*).mp |
| 8 | exp child/ or exp child care/ |
| 9 | exp newborn/ or exp newborn care/ |
| 10 | exp infant care/ or exp infant/ |
| 11 | exp juvenile/ |
| 12 | exp pediatrics/ |
| 13 | exp toddler/ |
| 14 | exp preschool child/ |
| 15 | 7 or 8 or 9 or 10 or 11 or 12 or 13 or 14 |
| 16 | exp “systematic review”/ or exp meta analysis/ or exp “review”/ |
| 17 | 6 and 15 and 16 |
